# National Well-Being Measures Before and During the COVID-19 Pandemic in Online Samples

**DOI:** 10.1007/s11606-020-06274-3

**Published:** 2020-10-19

**Authors:** Tyler J. VanderWeele, Jeffery Fulks, John F. Plake, Matthew T. Lee

**Affiliations:** 1 Department of Epidemiology, Harvard T. H. Chan School of Public Health, Boston, MA USA; 2 Human Flourishing Program, Institute for Quantitative Social Science, Harvard University, Cambridge, MA USA; 3 American Bible Society, Philadelphia, PA USA; 4 Evangel University, Springfield, MO USA

**Keywords:** well-being, flourishing, pandemic, COVID-19

## BACKGROUND

The COVID-19 pandemic has affected people’s lives in countless ways. The impact may extend to physical and mental health; their social relationships; their sense of meaning, identity, and happiness; and their financial stability.

## OBJECTIVE

Using a stratified online national sample, representative of the USA on geographic region, gender, generation/age, and race/ethnicity, we report on means of well-being scores in the USA across these various domains of human flourishing^1,2^ both prior to (January 2020) and following (June 2020) the WHO declaration of pandemic.

## METHODS AND FINDINGS

In January and June 2020, participants were recruited and surveyed through online Qualtrics national consumer panels (Lucid). The January study employed a 15-minute questionnaire. Data were collected from January 2 through 13, 2020 resulting in 1010 completed responses (completed-surveys-to-qualified-respondents rate, 90%) using a stratified national sample of adults 18 and older within all 50 states and the District of Columbia. Based on US Census data, quotas were designed to ensure that the final group of respondents reflected the distribution of adults nationwide and adequately represented the racial and ethnic diversity of the USA. Quotas limited responses by geographic region, gender, generation/age, and race/Hispanic-origin. No other screening criteria were applied. Post hoc weighting ensured the sample was representative of US adults in each quota area plus educational attainment and religious self-identification. Similar representative recruitment and online data collection was carried out in June 2020, from May 28 through June 10, 2020, resulting in 3020 completed responses (completed-surveys-to-qualified-respondents rate, 76%). Among the survey items, participants responded to 12 well-being items in six flourishing domains (happiness, health, meaning, character, relationships, financial; two indicators per domain) as part of an overall validated flourishing measure.^1,2^ Items were self-reported scored from zero to ten. Means at both time periods were reported across the six domains (Table 1), and *t* tests were used to assess changes in scores over time. Means for individual indicator scores at both time periods, and precise item wording, are reported in Table 2.

**Table 1:** Changes in Flourishing Domains in the USA from January 2020 to June 2020

Domain	January 2020 (*n* = 1010)	June 2020 (*n* = 3020)
	Mean (Std. Dev.)	Mean (Std. Dev.)
Happiness and life satisfaction	6.9 (2.1)	6.2 (2.3)
Mental and physical health	7.1 (2.0)	6.4 (2.2)
Meaning and purpose	7.0 (2.2)	6.6 (2.4)
Character and virtue	7.0 (1.8)	7.0 (2.0)
Close social relationships	6.9 (2.3)	6.7 (2.5)
Financial and material stability	5.7 (2.8)	4.8 (3.0)
Overall (secure flourishing index)	6.8 (1.7)	6.3 (1.7)

The secure flourishing index is an average score across the six domains including financial and material stability [2]. If the financial domain is omitted, then the resulting mean flourishing index [2] and standard deviation would be 7.0 (1.7) January 2020 and 6.6 (2.0) in June 2020

**Table 2:** Exact Item Wording, and Individual Indicator Means and Standard Deviations in January 2020 and June 2020

	January 2020 (*n* = 1010)		June 2020 (n = 3020)	
Item	Mean	Std. Deviation	Mean	Std. Deviation
Q1. Overall, how satisfied are you with life as a whole these days?	6.88	2.34	6.18	2.50
Q2. In general, how happy or unhappy do you usually feel?	6.92	2.18	6.15	2.33
Q3. In general, how would you rate your physical health?	6.76	2.14	6.19	2.39
Q4. How would you rate your overall mental health?	7.37	2.36	6.66	2.60
Q5. Overall, to what extent do you feel the things you do in your life are worthwhile?	7.15	2.24	6.68	2.43
Q6. I understand my purpose in life	6.82	2.57	6.50	2.78
Q7. I always act to promote good in all circumstances, even in difficult and challenging situations	7.21	2.11	7.19	2.26
Q8. I am always able to give up some happiness now for greater happiness later	6.78	2.19	6.75	2.24
Q9. I am content with my friendships and relationships	7.12	2.40	6.92	2.52
Q10. My relationships are as satisfying as I would want them to be	6.72	2.54	6.54	2.69
Q11. How often do you worry about being able to meet normal monthly living expenses?	5.73	3.17	5.00	3.20
Q12. How often do you worry about safety, food, or housing?	5.76	3.04	4.58	3.13
Flourishing Index	6.97	1.74	6.57	1.97
Secure Flourishing Index	6.77	1.66	6.28	1.66

† Each question or statement is evaluated 0-10. Anchors are^2^:

Q1 (0=Not Satisfied at All, 10=Completely Satisfied); Q2 (0=Extreme Unhappy, 10=Extremely Happy); Q3 and Q4 (0=Poor, 10=Excellent); Q5 (0=Not at All Worthwhile, 10=Completely Worthwhile); Q6, Q9, and Q10 (0=Strongly Disagree, 10=Strongly Agree); Q7 and Q8 (0=Not True of Me, 10=Completely True of Me); Q11 and Q12 (0=Worry All of the Time, 10=Do Not Ever Worry)

Q1 and Q2 constitute the Happiness & Life Satisfaction domain; Q3 and Q4 Mental & Physical Health; Q5 and Q6 Meaning & Purpose; Q7 and Q8 Character & Virtue; Q9 and Q10 Close Social Relationships; and Q11 and Q12 Financial and Material Stability. The Flourishing Index is an average of the responses from Q1 through Q10 and does not include the Financial and Material Stability domain; the Secure Flourishing Index is an average of the responses from Q1 through Q12 and does include the Financial and Material Stability domain.^2^ Reliability in cross-cultural samples is alpha=0.89 for the flourishing index and alpha=0.86 for the secure flourishing index.^2^ In the present sample, reliability in January is alpha=0.91 for the flourishing index, and alpha=0.81 for the secure flourishing; in June, reliability is alpha=0.93 for the flourishing index and alpha=0.86 for the secure flourishing index

In January 2020, mean scores were approximately seven in each domain, except for financial stability which was lower (Table 1). From January to June 2020, means in flourishing declined overall (− 0.49, 95% CI − 0.61, − 0.37, *p* < 0.001) and in every domain, except character. The declines were larger for self-reported health (− 0.64, 95% CI − 0.78, − 0.49, *p* < 0.001), happiness (− 0.74, 95% CI − 0.89, − 0.58, *p* < 0.001), and financial stability (− 0.95, 95% CI − 1.15, − 0.75, *p* < 0.001) than for social relationships (− 0.19, 95% CI − 0.36, − 0.02, *p* = 0.02), meaning and purpose (− 0.39, 95% CI − 0.55, − 0.23, *p* < 0.001), or character strengths (− 0.03, 95% CI − 0.16, 0.11, *p* = 0.68).

## DISCUSSION

Well-being has declined in the USA during the COVID-19 pandemic, but not all aspects have been affected equally. The health, happiness, and financial stability means each declined by about one-third of a standard deviation (e.g., a change from the 50th^-^percentile to 37th percentile of the original distribution). The modest declines in social connectedness scores, but more substantial declines in happiness and mental health corroborate recent evidence of only modest increases in loneliness but larger increases in psychological distress between 2018 and 2020.^3^ Compared with that prior data, the results here concern a much tighter time frame around the pandemic, and additional domains of well-being. The modest changes in meaning and character may be indicative of the human capacity to find growth amidst difficulty. That the financial stability scores manifested the largest decline likely mirrors the dramatic increase in unemployment and in employment insecurity, and may have subsequent health consequences.^4^

The study is limited by the sampling methodology, the online assessment requiring internet access and literacy, potential seasonality of well-being, and the possibility of selection bias since respondents in June were less likely to participate than in January. However, if less well-off individuals were less likely to respond in June, the declines in well-being may be even larger than those reported here.^5^ Likewise, while seasonality of subjective well-being sometimes indicates slightly lower levels in colder months, this too would then imply that the actual declines in well-being may be larger than those reported in Table 1.

Assessment of these flourishing domains has recently been proposed for use in clinical settings.^1^ The data here provide the first national benchmarks for these flourishing domains. These benchmarks may be of interest in clinical and public health assessment, and may also be of use in determining when the USA has returned to its prior levels of well-being.
